# Intensity of pyrethroid resistance in *Anopheles gambiae* before and after a mass distribution of insecticide-treated nets in Kinshasa and in 11 provinces of the Democratic Republic of Congo

**DOI:** 10.1186/s12936-020-03240-6

**Published:** 2020-04-30

**Authors:** Francis Wat’senga, Fiacre Agossa, Emile Z. Manzambi, Gillon Illombe, Tania Mapangulu, Tamfum Muyembe, Tiffany Clark, Mame Niang, Ferdinand Ntoya, Aboubacar Sadou, Mateusz Plucinski, Yikun Li, Louisa A. Messenger, Christen Fornadel, Richard M. Oxborough, Seth R. Irish

**Affiliations:** 1 Institut National de Recherche Biomédicale, PO Box 1192 Kinshasa, Democratic Republic of Congo; 2 USAID President’s Malaria Initiative, VectorLink Project, Abt Associates, 6130 Executive Blvd, Rockville, MD 20852 USA; 3 U.S. President’s Malaria Initiative, U.S. Agency for International Development, Kinshasa, Democratic Republic of the Congo; 4 U.S. President’s Malaria Initiative and Centers for Disease Control and Prevention, 1600 Clifton Road NE, Atlanta, GA 30329 USA; 5 London School of Hygiene and Tropical Medicine, Keppel Street, London, WC1E 7HT UK; 6 U.S. President’s Malaria Initiative, United States Agency for International Development, Bureau for Global Health, Office of Infectious Disease, 2100 Crystal Drive, Arlington, VA 22202 USA

**Keywords:** Pyrethroid, Resistance intensity, Democratic Republic of Congo, *Anopheles gambiae*, CDC bottle bioassay, WHO susceptibility test

## Abstract

**Background:**

Between 2011 and 2018, an estimated 134.8 million pyrethroid-treated long-lasting insecticidal nets (LLINs) were distributed nationwide in the Democratic Republic of Congo (DRC) for malaria control. Pyrethroid resistance has developed in DRC in recent years, but the intensity of resistance and impact on LLIN efficacy was not known. Therefore, the intensity of resistance of *Anopheles gambiae* sensu lato (s.l.) to permethrin and deltamethrin was monitored before and after a mass distribution of LLINs in Kinshasa in December 2016, and in 6 other sites across the country in 2017 and 11 sites in 2018.

**Methods:**

In Kinshasa, CDC bottle bioassays using 1, 2, 5, and 10 times the diagnostic dose of permethrin and deltamethrin were conducted using *An. gambiae* s.l. collected as larvae and reared to adults. Bioassays were conducted in four sites in Kinshasa province 6 months before a mass distribution of deltamethrin-treated LLINs and then two, six, and 10 months after the distribution. One site in neighbouring Kongo Central province was used as a control (no mass campaign of LLIN distribution during the study). Nationwide intensity assays were conducted in six sites in 2017 using CDC bottle bioassays and in 11 sites in 2018 using WHO intensity assays. A sub-sample of *An. gambiae* s.l. was tested by PCR to determine species composition and frequency of *kdr*-1014F and 1014S alleles.

**Results:**

In June 2016, before LLIN distribution, permethrin resistance intensity was high in Kinshasa; the mean mortality rate was 43% at the 5× concentration and 73% at the 10× concentration. Bioassays at 3 time points after LLIN distribution showed considerable variation by site and time and there was no consistent evidence for an increase in pyrethroid resistance intensity compared to the neighbouring control site. Tests of *An. gambiae* s.l. in 6 sites across the country in 2017 and 11 sites in 2018 showed all populations were resistant to the diagnostic doses of 3 pyrethroids. In 2018, the intensity of resistance varied by site, but was generally moderate for all three pyrethroids, with survivors at ×5 the diagnostic dose. *Anopheles gambiae* sensu stricto (s.s.) was the most common species identified across 11 sites in DRC, but in Kinshasa, *An. gambiae* s.s. (91%) and *Anopheles coluzzii* (8%) were sympatric.

**Conclusions:**

Moderate or high intensity pyrethroid resistance was detected nationwide in DRC and is a serious threat to sustained malaria control with pyrethroid LLINs. Next generation nets (PBO nets or bi-treated nets) should be considered for mass distribution.

## Background

Malaria remains the leading cause of consultation, hospitalization, and death in the Democratic Republic of Congo (DRC), with on average more than 5000 malaria deaths per month [1]. The National Malaria Control Programme (NMCP) has a strategic goal of protecting at least 80% of the population at risk with preventative measures by 2020 [2]. The primary vector control method used to protect people in DRC is the free distribution of long-lasting insecticidal nets (LLINs). LLINs have been distributed on a provincial level, with rolling mass distributions scheduled for provinces approximately every 3 years, and routine distribution being done through ante-natal clinic (ANC) visits, expanded programme of immunization (EPI) visits, and in some provinces, school-based distribution.

Pyrethroids have been the insecticide class of choice for mosquito nets for more than 30 years and are still an important component of every LLIN that currently has prequalified status by World Health Organization (WHO) [3]. These compounds are fast-acting, safe for human contact, and have shown impressive community-level effects when deployed in areas where malaria vectors are susceptible [4, 5]. Pyrethroid resistance was first reported in malaria vectors in West Africa in the 1980s and 1990s [6, 7] and has since become widespread across most of sub-Saharan Africa [8]. The implications for malaria vector control are not clear, but resistance is a serious concern to the WHO [9] and the NMCP of DRC [10], with fears that pyrethroid resistance may compromise the efficacy of pyrethroid LLINs. Despite this, a number of studies in sub-Saharan Africa have shown that LLINs continued to help reduce malaria cases even with the presence of pyrethroid resistant malaria vectors [11–15].

In DRC, studies from 2013 have shown no significant difference in the odds of malaria infection between people owning a permethrin LLIN and those without a net, while those with deltamethrin- and alpha-cypermethrin-treated nets had significantly reduced odds of malaria infection [16, 17]. These results corresponded with the higher frequency of permethrin resistance than resistance to deltamethrin noted in susceptibility tests conducted in 2013 [18] (MPSMRM, 2014). Molecular analysis of pyrethroid resistant *Anopheles* malaria vectors in several locations in DRC has shown the upregulation of genes related to metabolic resistance that were associated with high rates of *Plasmodium* infection and loss of LLIN efficacy [19, 20].

Previous published susceptibility data from DRC has focused on the use of a diagnostic concentration of insecticide to determine whether a mosquito population is susceptible or resistant [9, 18, 21]. However, it is thought that the intensity of pyrethroid resistance may be an important indicator of potential pyrethroid LLIN control failure [22, 23]. For this reason, annual insecticide resistance intensity testing has been scaled-up in DRC [18, 21]. While agricultural use of pyrethroids has been associated with initial development of resistance in some studies [24, 25], other studies have found that mass distribution of LLINs was associated with increasing resistance [26–28]. These studies have mostly looked at mosquito populations retrospectively and little is known about how rapidly these changes occur following a LLIN distribution campaign. Therefore, part of this study was to monitor pyrethroid resistance intensity in suburbs of Kinshasa before and after mass LLIN distribution in December 2016. Additionally, intensity of pyrethroid resistance was monitored nationwide in six other sites in 2017 and 11 sites in 2018.

## Methods

### Study sites

**Fig. 1:**
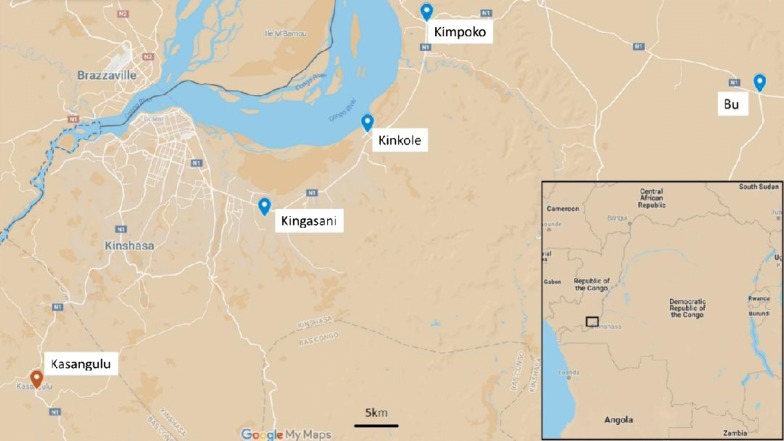
Sites where *Anopheles gambiae* s.l. were collected for pyrethroid intensity bio-assays in Kinshasa Province (Kingasani, Kinkole, Kimpoko and Bu) as well as Kasangulu site in neighbouring Kongo Central Province

The first part of the study was conducted in Kinshasa Province in 2016 and 2017. Four sites were selected for mosquito larval collection to monitor changes in *Anopheles gambiae* sensu lato (s.l.) pyrethroid resistance intensity following mass LLIN (DawaPlus 2.0 coated with deltamethrin at a target dose of 80 mg/m^2^) distribution in December 2016 (Fig. 1). A fifth site, Kasangulu, in neighbouring Kongo Central province was selected to provide a “control” site, which would not be included in the Kinshasa Province LLIN distribution campaign in 2016. However, a limitation is that PermaNet 2.0 LLINs (formulation of deltamethrin at a target dose of 55 mg/m^2^)) were distributed in a mass campaign in Kongo Central Province in 2014. More details of the study sites and previous mass LLIN distributions in each region are presented in Additional file 1: Table S1.

**Fig. 2:**
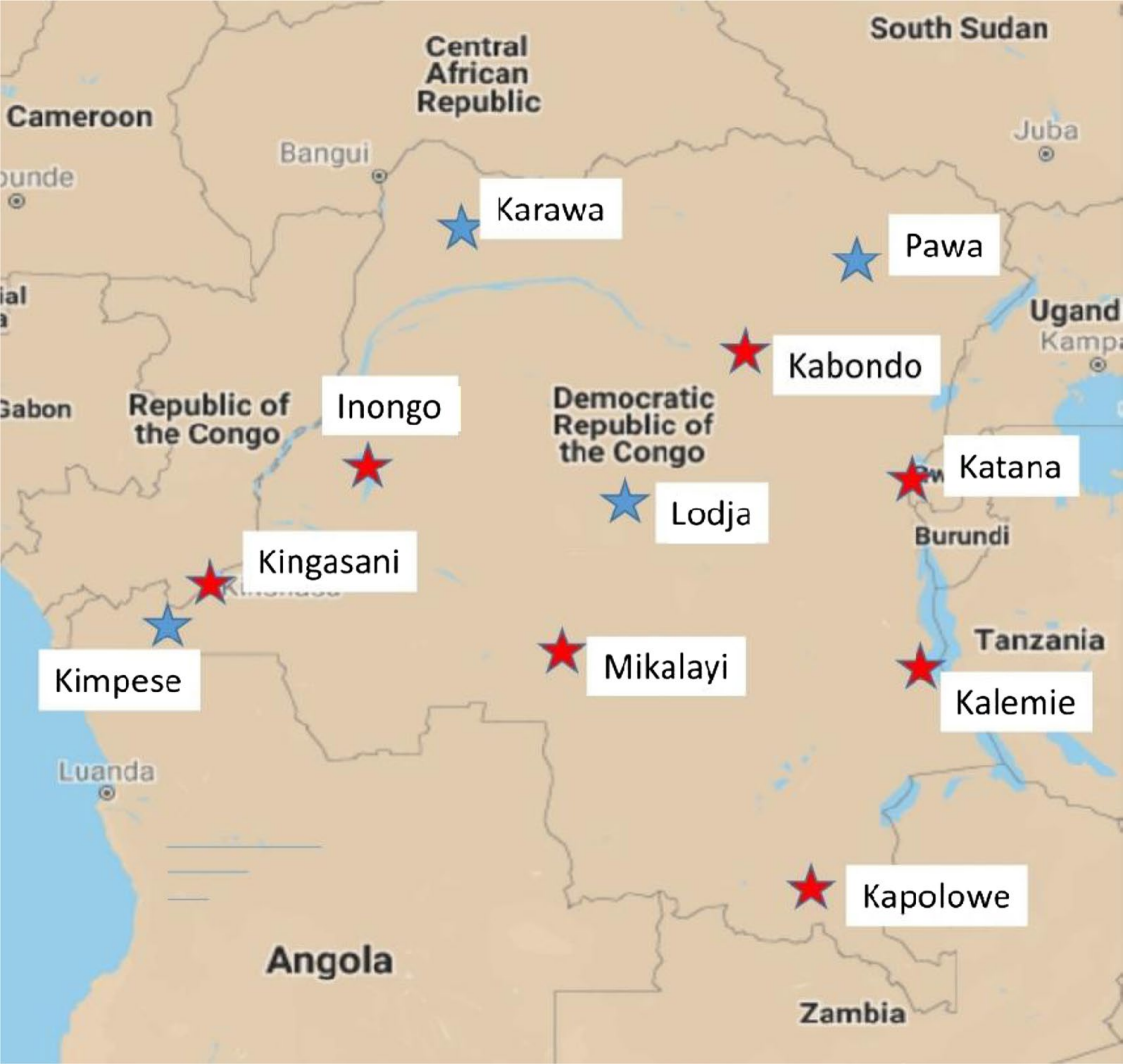
Sites where pyrethroid intensity assays were conducted throughout DRC in 2017 and 2018. Red stars indicate sites where bio-assays were conducted in 2017 and 2018; blue stars indicate sites where bio-assays were conducted in 2018 only. Note that the 2017 Kingasani results are presented with the Kinshasa results

The second part of the study involved nationwide testing of pyrethroid resistance intensity. Deltamethrin and permethrin resistance intensity tests were conducted in six sites in 2017 using Centers for Disease Control and Prevention (CDC) bottle bioassays. In 2018, testing was expanded to eleven sites, with resistance intensity to permethrin, alpha-cypermethrin and deltamethrin monitored using WHO tube tests for intensity (Fig. 2). More site details are included in Additional file 1: Table S1.

### Insecticide susceptibility tests

Mass LLIN distribution took place in December 2016 in Kinshasa Province. CDC intensity assays were conducted once before the mass distribution of LLINs (June 2016) and two, six, and ten months after the distribution (February 2017, June 2017, and October 2017). For each round of bioassays, *An. gambiae* s.l. larvae were collected from the five sites (Fig. 1) and returned to the laboratory at the *Institute National de Recherche Biomédicale* (INRB) in Kinshasa city, where they were reared to adults. Adult mosquitoes were kept in cages and provided with 10% sugar solution ad libitum until the time of testing at the age of 2–5 days.

The intensity assays conducted nationwide followed the same protocol, but tests were conducted once per year (all tests between January and August in 2017 and 2018) and mosquitoes were reared and tested in field insectaries.

### CDC bottle bioassays

CDC bottle bioassays were conducted to determine the intensity of pyrethroid resistance, following standard guidelines [29, 30]. Pre-measured vials of technical grade active ingredient were supplied by CDC and made into stock solutions for each insecticide dose by diluting with acetone. Stock solutions were stored in the refrigerator (4 °C) in light-proof bottles for future use. Glass Wheaton bottles (250 ml) were washed with warm soapy water and rinsed thoroughly with water at least three times and left to dry overnight. A disposable pipette was used to transfer 1 ml of acetone into the negative control bottle and 1 ml of each stock solution into the respective treatment bottle. Bottles were swirled so that the glass bottom and inside cap were coated before being placed on their side and rotated while rocking so that the sides were evenly coated with insecticide. The bottles were protected from sunlight, and caps removed before being left to dry overnight.

An aspirator was used to gently add twenty-five mosquitoes into each bottle per replicate. Four replicates of each dose were done to reach approximately 100 mosquitoes tested. Mosquitoes were exposed for the diagnostic time of 30 min, with knock-down being recorded at the end of exposure. A knocked-down mosquito was defined as not being able to stand. Deltamethrin was tested at 1× (12.5 μg/bottle), 2× (25 μg/bottle), 5× (62.5 µg/bottle), and 10× (125 µg/bottle) the diagnostic dose for *Anopheles*. Permethrin was also tested at 1× (21.5 μg/bottle), 2× (43 μg/bottle), 5× (107.5 µg/bottle), and 10 times (215 µg/bottle) the diagnostic dose for *Anopheles*.

### WHO susceptibility tests

In 2018, insecticide susceptibility and resistance intensity testing were conducted in 11 sentinel sites (Fig. 1) using the WHO tube test. The insecticides tested in 2018 were: deltamethrin 1×, 5×, 10× (0.05%, 0.25%, 0.5%); permethrin 1×, 5×, 10× (0.75%, 3.75%, 7.5%) and alpha-cypermethrin 1×, 5×, 10× (0.05%, 0.25%, 0.5%). In all sites, susceptibility testing was conducted with adult *An. gambiae* s.l., following WHO protocols [22]. INRB entomologists traveled to each site to collect larvae and pupae, which were reared to female adult mosquitoes aged 2–5 days and exposed for 1 hour to insecticide-treated filter papers provided by the WHO (prepared by Universiti Sains Malaysia). All tests were accompanied by negative control tests where mosquitoes were exposed to filter papers impregnated with oil or solvent. Testing was done according to WHO protocols, with mortality read 24 h after exposure. Four replicates of 25 *An. gambiae* s.l. were exposed to each concentration.

### Identification of species and target site mutations

A subset of *An. gambiae* s.l. that were collected from the four sites in Kinshasa (Bu, Kimpoko, Kingasani and Kinkole) and 1 ‘control’ site in Kongo Central (Kasungulu) in October 2017 and tested in CDC control bottles, were sent to CDC, Atlanta, USA for molecular analysis. In addition, 100 mosquitoes used for WHO bioassays in each of the eleven nationwide sites in 2018 were used for molecular analysis at INRB, Kinshasa, DRC. PCR was used to determine the species of mosquitoes from the *An. gambiae* complex and to determine the frequency of the voltage-gated sodium channel mutation (VGSC) 1014S (formerly known as *kdr*-*east*) and VGSC-1014F (formerly known as *kdr*-*west*).

Genomic DNA was extracted from whole mosquitoes at CDC using Extracta^TM^ DNA Prep for PCR-Tissue kits (QuantaBio, USA) and at INRB using the CTAB method [31]. Species identification was performed according to the protocol of Wilkins et al. [32] at CDC and using the protocol of Santolamazza et al. [33] at INRB. The VGSC-1014S and 1014F alleles were detected using adapted protocols for allele-specific PCR (AS-PCR) [34–36]. *Anopheles coluzzii* AKDR and *An. gambiae* sensu stricto (s.s.) RSP-ST strains from the Malaria Research and Reference Reagent Resource Center (MR4), were used as positive controls, alongside negative (no template) controls.

### Analysis

Scoring of bottle bioassays using the diagnostic dose followed WHO and CDC criteria, with mortality of 98–100% indicating susceptibility, 90–97% indicating possible resistance that should be confirmed, and less than 90% indicating resistance [22, 29]. Mortality of 98–100% at the 5× concentration (but < 90% at 1×) indicates low resistance intensity. Mortality < 98% at the 5 × concentration but 98–100% at the 10× concentration indicates moderate resistance intensity. Mortality < 98% at the 10× concentration indicates high resistance intensity [22].

The comparison of bioassay results prior to the LLIN mass distribution and after distribution in Kinshasa were made using a logistic regression model, taking into account the dose, site, time period, and an interaction between dose and site as fixed effects and bottle as a random effect. Analysis was done using the glmm function in R (version 3.2.3). Pearson’s Chi squared test was used to determine deviations from Hardy–Weinberg equilibrium for VGSC-1014F allele frequencies.

## Results

### Intensity of resistance in Kinshasa Province before and after LLIN mass distribution using CDC bottle bioassay

**Fig. 3:**
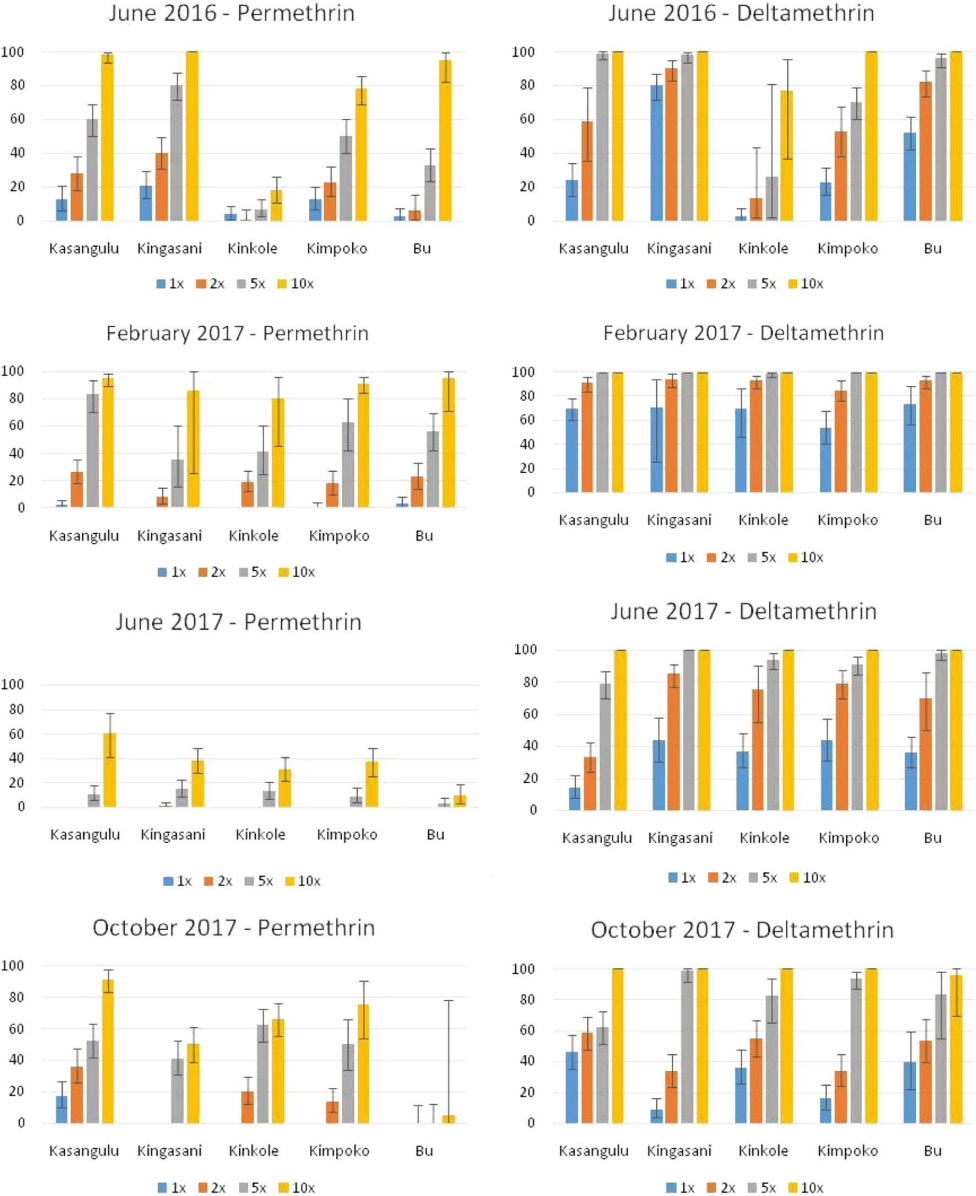
Adjusted estimates (and 95% confidence intervals) for mortality (at 30 min) of *An. gambiae* s.l. from Kinshasa province in CDC bottle intensity assays conducted in 2016–2017

Over the four periods of testing, a total of 15,200 *An. gambiae* s.l. were used for resistance intensity bioassays in Kinshasa Province. Resistance to permethrin and deltamethrin was found in all sites (Fig. 3). In June 2016, before LLIN distribution mean results for Kinshasa (mean of Kingasani, Kinkole, Kimpoko, Bu) showed that permethrin resistance intensity was high and the mean mortality rate was 43% at the 5× concentration and 73% at the 10× concentration. After the mass distribution of LLINs in December 2016 (mean results for February, June and October tests) the mean mortality rate in Kinshasa was 32% for 5× and 60% with permethrin at the 10× concentration. The mean resistance intensity to deltamethrin was also high before LLIN distribution (75% at 5× and 94% mortality at 10× concentration) but decreased after LLIN distribution to a mean of 95% and 99% mortality at 5 and 10× concentrations, respectively. In general, levels of resistance were lower for deltamethrin, compared to permethrin. However, there was considerable variation in the results by site (Table 1). The hypothesis was that resistance intensity to permethrin and deltamethrin would increase in Kinshasa following LLIN distribution, compared to the control site of Kasangulu. In the control site of Kasangulu, mortality in permethrin intensity tests decreased significantly in 2017 (indicating an increase in resistance).

**Table 1:** Odds ratios and p-values for permethrin and deltamethrin susceptibility bioassays conducted in and around Kinshasa, DRC, taking into account dose, site, evaluation period, and the change in the distribution site relative to the control site

	Permethrin		Deltamethrin	
	Odds ratio	p-value	Odds ratio	p-value
Dose				
1×	Reference		Reference	
2×	3.44	< 0.001	2.49	< 0.001
5×	13.61	< 0.001	10.48	< 0.001
10×	79.97	< 0.001	Undefined	< 0.001
Site				
Kasangulu	Reference		Reference	
Bu	0.23	0.003	2.27	0.010
Kimpoko	0.47	0.045	0.42	0.008
Kingasani	1.62	0.153	5.75	< 0.001
Kinkole	0.02	< 0.001	0.07	< 0.001
Evaluation period				
Pre-distribution	Reference		Reference	
Post-distribution	0.49	< 0.001	1.09	0.568
Change in distribution site relative to the control site (Kasangulu)				
Bu	0.70	0.099	0.66	0.072
Kimpoko	0.95	0.791	2.57	< 0.001
Kingasani	0.11	< 0.001	0.21	< 0.001
Kinkole	11.49	< 0.001	22.00	< 0.001

Resistance intensity was greater in Kinkole for permethrin (OR 11.49, p-value < 0.001) and deltamethrin (OR 22.00, p-value < 0.001) compared to Kasangulu post-LLIN distribution and also in Kimpoko for deltamethrin (OR 2.57, p-value < 0.001). In Kingasani, the opposite trend was recorded with a significantly lower resistance intensity following LLIN distribution for permethrin (OR 0.11, p-value < 0.001) and deltamethrin (OR 0.21, p-value < 0.001) compared to the control site, while in Bu there was no significant change post-distribution in resistance intensity for either insecticide.

### Intensity of permethrin and deltamethrin resistance in six sites in DRC in 2017 using CDC bottle bioassay

**Fig. 4:**
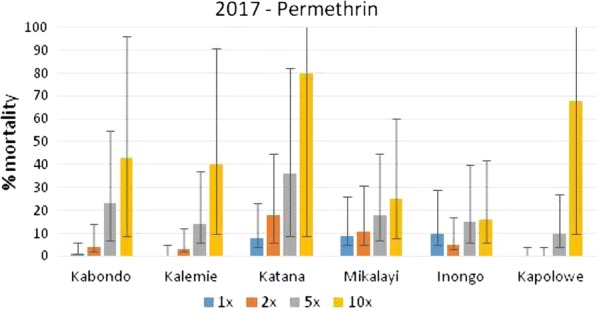
Mortality (and 95% confidence intervals) of wild *Anopheles gambiae* s.l. collected in six sites in DRC and tested in CDC intensity assays with permethrin

Nationwide bioassay testing showed that permethrin resistance was present in all 6 sites, with less than 10% mortality at the diagnostic dose. Mortality rates increased slightly with increased concentration, but high intensity permethrin resistance was present in all sites, with considerably less than 98% mortality at 10× the diagnostic concentration of permethrin (Fig. 4).

**Fig. 5:**
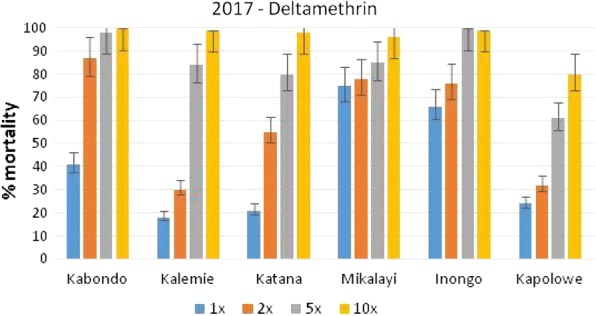
Mortality (and 95% confidence intervals) of wild *Anopheles gambiae* s.l. collected in six sites in DRC and tested in CDC intensity assays with deltamethrin

*Anopheles gambiae* s.l. populations were less intensely resistant to deltamethrin, although all populations tested were resistant at 1× and 2× the diagnostic dose. The resistance intensity was low (> 98% mortality at 5× dose) in Kabondo and Inongo, moderate in Kalemie and Katana (> 98% mortality at 10× dose) and high (< 98% mortality at 10× dose) in Mikalayi and Kapolowe (Fig. 5).

### Intensity of permethrin, deltamethrin and alpha-cypermethrin resistance in eleven sites in 2018 in DRC using the WHO tube test

**Fig. 6:**
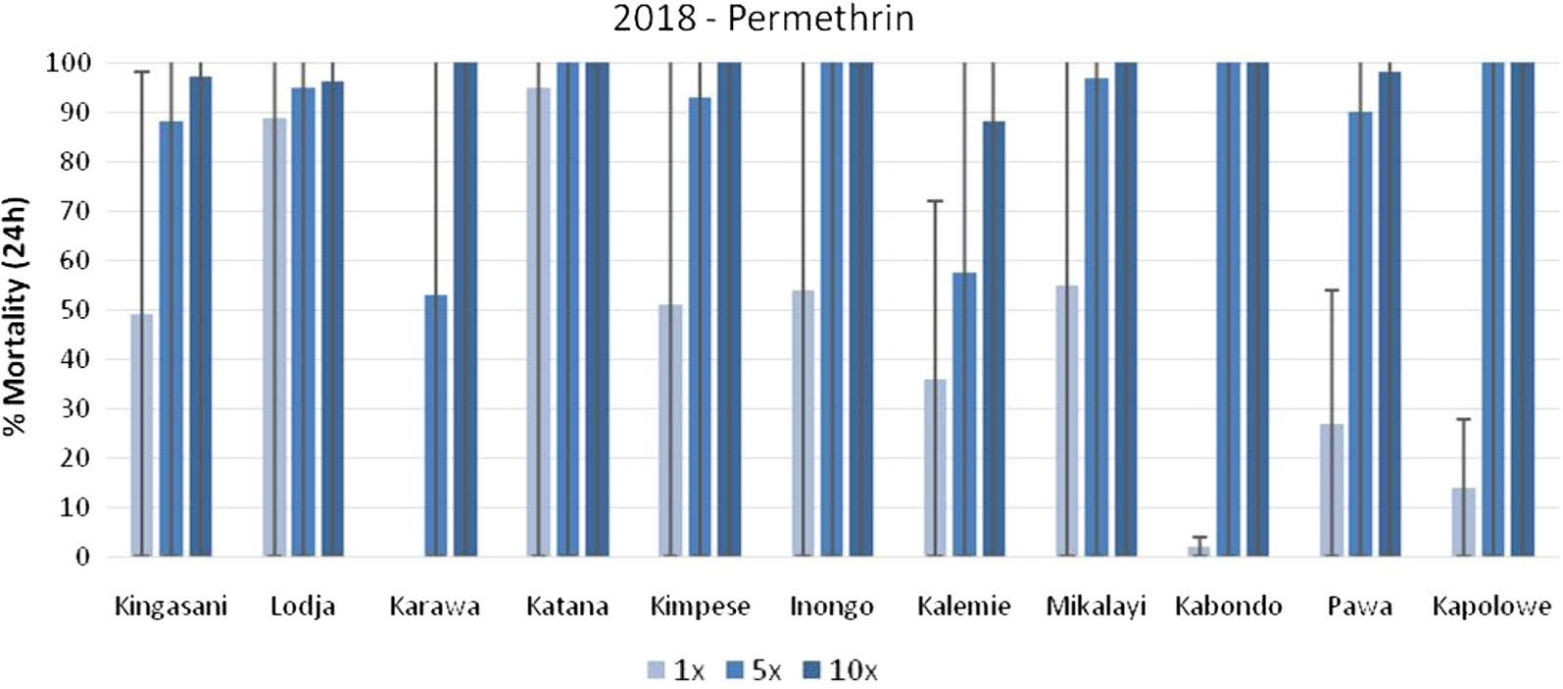
Percentage mortality of *An. gambiae* s.l. after exposure to permethrin at 1×, 5×, and 10× times the diagnostic concentration.*In Karawa, 0% mortality was recorded with permethrin 1×

**Fig. 7:**
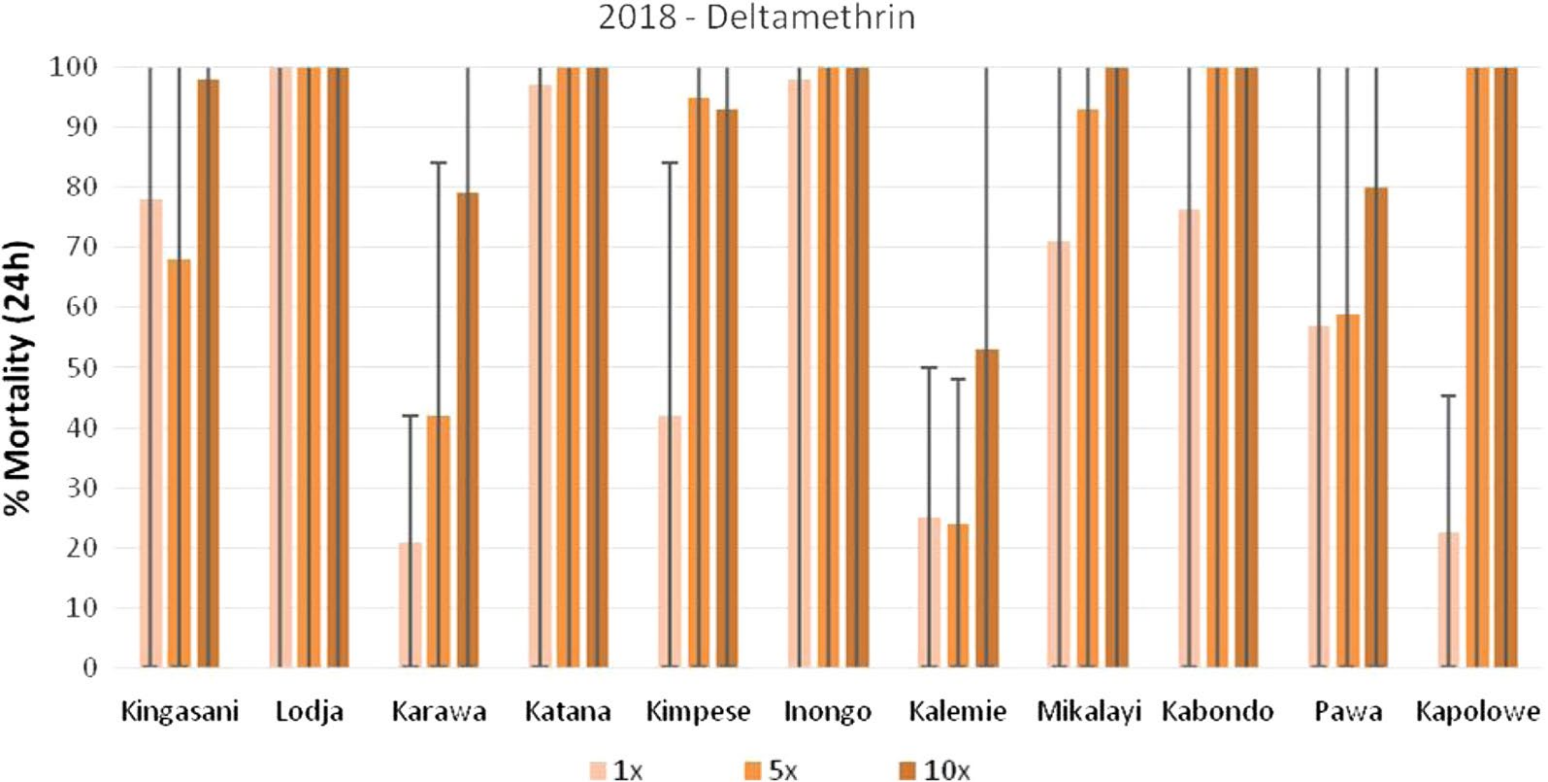
Percentage mortality of *An. gambiae* s.l. after exposure to deltamethrin at 1×, 5×, and 10× times the diagnostic concentration

**Fig. 8:**
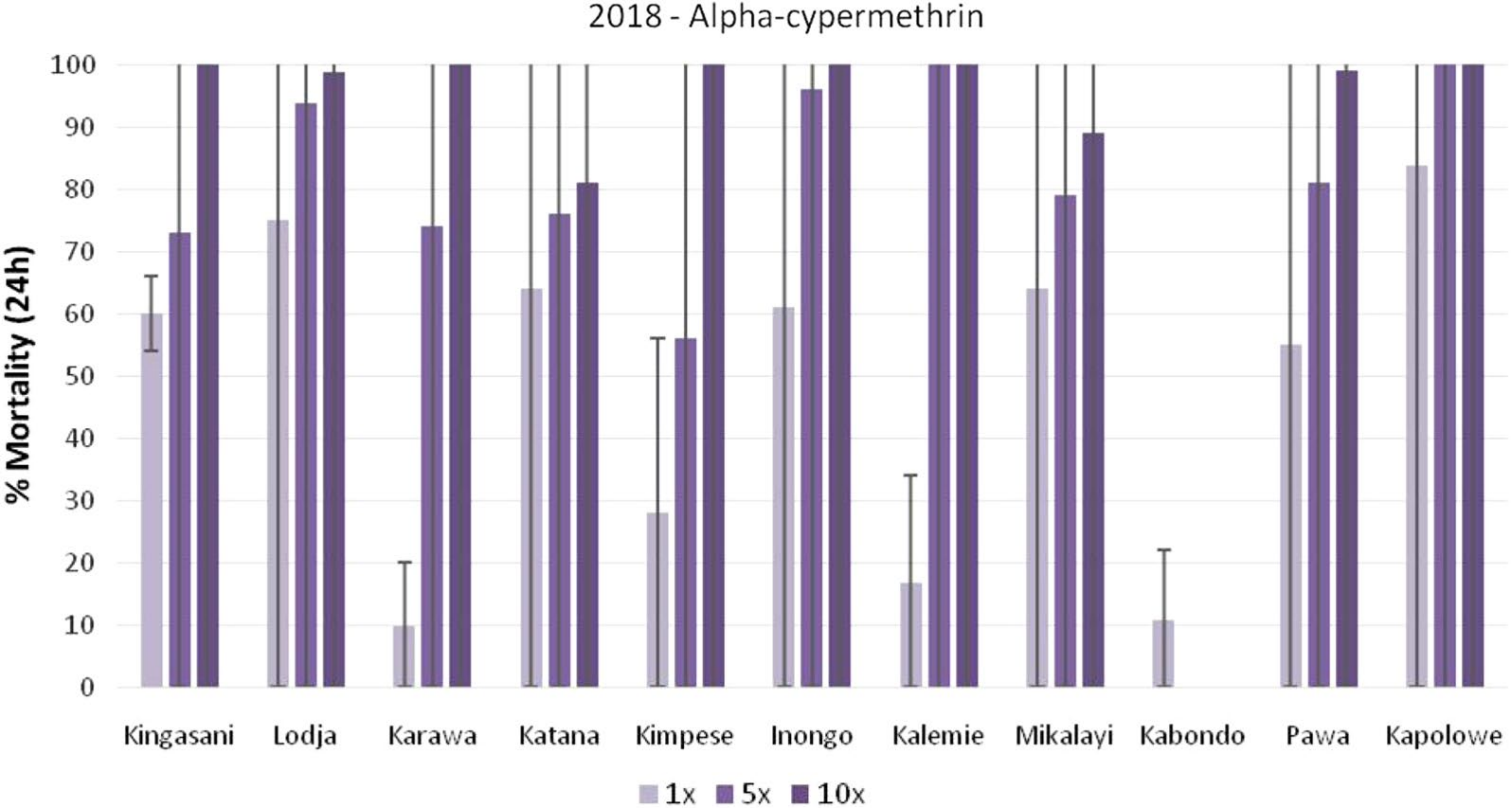
Percentage mortality of *An. gambiae* s.l. after exposure to alpha-cypermethrin at 1×, 5×, and 10× times the diagnostic concentration.*In Kabondo, alpha-cypermethrin 0.25% and 0.5% were not tested

In 2018, nationwide WHO insecticide susceptibility and resistance intensity tests were completed with *An. gambiae* s.l. populations in 11 sites. The data is presented in Figs. 6, 7, and 8 for permethrin, deltamethrin and alpha-cypermethrin, respectively. In Kabondo, testing with alpha-cypermethrin 5× and 10× was not completed as mortality was > 20% in the control and the field team was unable to find sufficient larvae for repeat tests. Resistance to permethrin (< 90% mortality) was observed in all sites at the diagnostic dose (1×), except Katana, where there was possible resistance (90–98% mortality). Resistance intensity was low in Katana, Inongo, and Kapolowe; moderate (> 98% mortality at 5× dose) in Karawa, Kimpese, Mikalayi, and Pawa; and high (< 98% mortality at 10× dose) in Kingasani, Lodja, and Kalemie (Fig. 6).

Resistance to deltamethrin was recorded in all sites, except Katana (possible resistance), Lodja and Inongo (susceptible). The intensity of resistance was low in Kapolowe and Kabondo, moderate in Mikalayi, and high in Kingasani, Karawa, Kimpese, Kalemie, and Pawa (Fig. 6).

Resistance to alpha-cypermethrin was also observed in all sites. The intensity was low in Kalemie and Kapolowe, high in Katana, Mikalayi, and Lodja, and moderate in the remaining five sites (Fig. 7).

### Species identification within the *An. gambiae* complex and frequency of VGSC-1014F and 1014S resistance alleles

#### Kinshasa resistance intensity study post-LLIN distribution (2017)

A total of 217 *An. gambiae* s.l. were tested for species identification and of those samples, 189 were analysed for VGSC-1014F and 1014S frequency. The primary species found was *An. gambiae* s.s. (91%). *Anopheles coluzzii* were only identified in Kimpoko (9%) and Kinkole (32%). Hybrid *An. gambiae* s.s./*An. coluzzii* were only found in Kinkole (2/44) (Table 2). One percent of samples did not amplify.

**Table 2:** Species identification within the complex *An. gambiae* s.l. from study sites in the province of Kinshasa

Site	*An. gambiae* species n(%)			
	*An. gambiae*s.s.	*An. coluzzii*	Hybrid	Total
Bu	32 (100%)	0	0	32 (100%)
Kasangulu	46 (100%)	0	0	46 (100%)
Kimpoko	30 (91%)	3 (9%)	0	33 (100%)
Kingasani	62 (100%)	0	0	62 (100%)
Kinkole	28 (64%)	14 (32%)	2 (5%)	44 (100%)
Total	198 (91%)	17 (8%)	2 (1%)	217 (100%)

Overall, both *An. gambiae* s.s. and *An. coluzzii* carried VGSC-1014F and 1014S resistance alleles, albeit at different frequencies. The mean frequency of resistance alleles determined across all sites for *An. gambiae* s.s. was 83% for homozygote VGSC-1014F, 3% for homozygote 1014S and 14% for heterozygous 1014F/1014S (Table 3). All VGSC-1014F frequencies were over 70% for *An. gambiae* s.s. and the highest was observed in Kingasani and Kinkole (both 91%). The trend was different for *An. coluzzii*, with 94% (16/17) being homozygous for the VGSC-1014S allele.

**Table 3:** *Kdr L1014F* and *L1014S* resistance alleles from the study sites in the province of Kinshasa

*Anopheles* species	Site	Homozygous *kdr*-*west* (L1014F/L1014F)	Homozygous *kdr*-*east* (L1014S/L1014S)	Heterozygous -*west/*-*east* (L1014F/L1014S)
*Anopheles gambiae* s.s.	Kasangulu	28 (0.70)	3 (0.08)	9 (0.23)
	Kingasani	52 (0.91)	1 (0.02)	4 (0.07)
	Kinkole	21 (0.91)	1 (0.04)	1 (0.04)
	Kimpoko	18 (0.86)	0 (0.00)	3 (0.14)
	Bu	21 (0.78)	0 (0.00)	6 (0.22)
Total *An. gambiae* s.s.	All sites	140 (0.83)	5 (0.03)	23 (0.14)
*An. coluzzii*	Kinkole	0 (0.00)	14 (1.00)	0 (0.00)
	Kimpoko	1 (0.33)	2 (0.67)	0 (0.00)
Total *An. coluzzii*	All sites	1 (0.06)	16 (0.94)	0 (0.00)
*Anopheles gambiae* s.s.*/An. coluzzii*	Kinkole	0 (0.00)	0 (0.00)	2 (1.00)
*An. gambiae* s.l.	Overall	141 (0.75)	21 (0.11)	25 (0.13)

#### Resistance intensity survey covering 11 sites nationwide (2018)

A total of 998/1100 (91%) *An. gambiae* s.l. were successfully amplified for species identification and 862/1100 (78%) for L1014F. Overall, *An. gambiae* s.s. (98.5%) was the primary species in all 11 sites. *Anopheles coluzzii* were only found in Kingasani (1%) and Mikalayi (1%) and 1% of hybrid *An. gambiae* s.s./*An. coluzzii* were found in Kingasani (Table 4). The VGSC-1014F frequency for *An. gambiae* s.l. recorded over eleven sites in 2018 varied between 0.85 (Kingasani) and 1.0 (Pawa and Karawa). The mean VGSC-1014F was close to fixation at 0.98 (Table 5). Evidence for significant deviations from Hardy–Weinberg equilibrium were observed for VGSC-1014F in Kingasani, Kalemie, Kabondo and Katana (Table 5).

**Table 4:** Species identification within the complex *An. gambiae* s.l. over eleven study sites in 2018

Site	*An. gambiae* species n (%)			Total	Did not amplify
	*An. gambiae* s.s.	*An. coluzzii*	Hybrid		
Lodja	65 (65%)	0	0	100	35 (35%)
Kapolowe	88 (88%)	0	0	100	12 (12%)
Kingasani	82 (82%)	4 (4%)	3 (3%)	100	11 (11%)
Mikalayi	53 (53%)	8 (8%)	0	100	39 (39%)
Kalemie	96 (96%)	0	0	100	4 (4%)
Kimpese	99 (99%)	0	0	100	1 (1%)
Pawa	100 (100%)	0	0	100	0
Karawa	100 (100%)	0	0	100	0
Inongo	100 (100%)	0	0	100	0
Kabondo	100 (100%)	0	0	100	0
Katana	100 (100%)	0	0	100	0
Overall	983 (89%)	12 (1%)	3 (1%)	1100	102 (9%)

**Table 5:** *L1014F* resistance alleles over eleven study sites in 2018

Site	Number tested	*RR*	*RS*	SS	Did not amplify	Frequency 1014F	χ^2^	p-value
Lodja	100	98	1	0	1	0.99	0.0026	0.96
Kapolowe	100	98	1	0	1	0.99	0.0026	0.96
Kingasani	100	64	0	11	25	0.85	61	< 0.000
Mikalayi	39	31	0	4	4	0.89	–	–
Kalemie	100	90	0	3	7	0.97	93	< 0.000
Kimpese	100	96	1	0	3	0.99	0.0026	0.96
Pawa	100	86	0	0	14	1	–	–
Karawa	100	95	0	0	5	1	–	–
Inongo	100	95	3	0	2	0.98	0.024	0.88
Kabondo	100	50	0	1	49	0.98	51	< 0.000
Katana	100	80	0	3	17	0.96	83	< 0.000
Overall	1039	883	6	22	128	0.97		

RR means homozygote resistant, RS means heterozygote resistant and SS means homozygote susceptible

## Discussion

Insecticide-treated nets are believed to be an important source of selection pressure for pyrethroid resistance genes in African malaria vectors [27, 37]. In addition to LLINs, other environmental factors such as agricultural pesticide run off into mosquito larval sites, may exert additional selection pressure on malaria vectors [24, 38]. Between 2011 and 2018, an estimated 134.8 million LLINs were distributed nationwide in DRC through mass campaigns and through routine distribution in schools and during ANC and EPI visits [39]. National Demographic and Health Surveys (DHS) have documented a substantial increase in net ownership, from just 9% of households nationwide owning at least one LLIN in 2007 [40], compared with 51% in 2010 [41] and 70% in 2013/14 [42]. This scale up of LLINs in DRC has coincided with the gradual spread of pyrethroid resistance and more recent increase in resistance intensity. Following a mass LLIN distribution campaign in Kinshasa in 2016, this study produced no consistent evidence for an increase in pyrethroid resistance intensity compared to the neighbouring control site of Kasungulu, where there was no mass LLIN campaign in 2016. There was a great deal of variation over time by insecticide and site. It is difficult to design a study to effectively measure the contribution of mosquito nets to selection pressure of mosquitoes, since LLINs are already widely distributed in DRC and pyrethroid resistance is prevalent in all malaria eco-epidemiological settings. It is also difficult to measure the impact of household and agricultural use of pyrethroids. Mass LLIN campaigns had previously been conducted in Kinshasa in 2008 and 2013, and in Kongo Central Province (where Kasungulu is situated) in 2012 and 2014 (Additional file 1: Table S1). Pyrethroid selection pressure had been ongoing for many years before the 2016 distribution in Kinshasa, which may explain the lack of difference between sites following the 2016 LLIN campaign in Kinshasa.

Nationwide tests of malaria vector populations in 6 sites in 2017 and 11 sites in 2018 showed all populations were resistant to diagnostic doses of type I (permethrin) and type II (deltamethrin and alpha-cypermethrin) pyrethroids. Regular monitoring of vector resistance has shown that pyrethroid resistance in *An. gambiae* s.l. became widespread in DRC relatively recently. Permethrin susceptible *An. gambiae* s.l. were present in Kinshasa in 2009 [21], while deltamethrin susceptibility was recorded in Lodja (Sankuru Province), Kalemie (Tanganyika Province), Kapolowe (Haut Katanga), Katana (Sud Kivu) and Kinshasa in 2016 [18]. Resistance to permethrin, deltamethrin and alpha-cypermethrin now appears to be present nationwide.

*Anopheles gambiae* s.s. was the most common vector species identified among the *An. gambiae* complex analysed across 11 sites in DRC. However, in Kinshasa, *An. gambiae* s.s. (91%) and *An. coluzzii* (8%) were sympatric and there was a small proportion of hybrid *An. gambiae* s.s./*An. coluzzii* (5%) in Kinkole. Though the frequency of hybrids in the *Anopheles* population from Kinkole is low, mating seems to be occurring between the two species. Both species are commonly sympatric in Central Africa, but hybrids of *An. gambiae* s.s./*An. coluzzii* are usually very uncommon [43, 44]. Populations of *An. gambiae* and *An. coluzzii* have previously been shown to be sympatric in several geographical areas in DRC, including Lodja, Mikalayi, Kalemie, Katana, Kinshasa, Kimpese and Inongo [18].

Both *An. gambiae* s.s. and *An. coluzzii* carried VGSC-1014F and 1014S alleles. However, *An. coluzzii* in Kinshasa had a high frequency of the 1014S allele, while *An. gambiae* s.s. had a high frequency of the 1014F allele; the latter observation may partially explain the higher levels of local permethrin resistance, despite deltamethrin-treated LLINs predominating in the most recent mass distribution campaigns. VGSC-1014F and L1014S are suspected to play a larger contributing role in resistance to type I (permethrin) versus type II (deltamethrin and alpha-cypermethrin) pyrethroids [45]. Interestingly, a proportion of heterozygous *An. gambiae* s.s. from Kinshasa harboured both VGSC-1014F and 1014S alleles. The phenomenon of these mutations co-occurring in individual mosquitoes has previously been reported in Senegal [46] and Uganda [47] and in Nord Ubangi, DRC [48]; however, the biological implications of possessing both resistance genotypes remain unknown and warrant further investigation. A limitation of this study is that only target site mutations for resistance were investigated. Mixed function oxidases (MFO) are implicated in pyrethroid resistance in several sites in DRC [49]. In addition, bioassays in 2016 showed increased mortality in permethrin resistant populations in DRC after pre-exposure to synergist piperonyl butoxide (PBO) [18]. The genetic basis conferring resistance to pyrethroids in malaria vectors *An. gambiae* s.s. and *An. coluzzii* needs to be investigated at the national level to improve malaria control decision-making, particular with regard to choice of LLINs for mass distribution campaigns.

Widespread pyrethroid resistance, particularly high intensity resistance, is of great importance for the NMCP for the implementation of evidence-based resistance management strategies and deployment of efficacious malaria vector control tools. Resistance intensity assays showed that neither 1, 5 or 10 times the diagnostic concentrations of permethrin, deltamethrin and alpha-cypermethrin were sufficient to provide adequate mortality of *An. gambiae* s.l. collected from 6 nationwide sites in 2017 tested using CDC bottle bioassays and 11 sites in 2018 using WHO tube tests. The WHO states that “when resistance is confirmed at the 5× and especially at the 10× concentrations, operational failure is likely” [22]. Pyrethroid LLINs should continue to offer some protection from malaria even in locations with high intensity resistance, through a combination of physical barrier, reduced survival of malaria vectors and malaria parasites [50–52]. However, next generation LLINs either impregnated with pyrethroids and the synergist PBO or containing chlorfenapyr (Interceptor G2^®^) are potential alternatives for the improved efficacy of LLINs and for resistance management. Several experimental hut studies have shown improved efficacy of PBO and chlorfenapyr LLINs in controlling pyrethroid resistant malaria vectors compared to conventional pyrethroid LLINs [53–57]. LLINs containing PBO or novel insecticide classes should be considered by the NMCP of DR Congo for future LLIN distribution campaigns in areas of moderate to high intensity of pyrethroid resistance, although the costs of these nets would also need to be considered.

## Conclusion

The widespread presence of moderate to high intensity pyrethroid resistance across all sentinel sites in DRC is a great concern. There was a great deal of variation in resistance over time by insecticide and no consistent evidence for an increase in pyrethroid resistance intensity was observed following the mass LLIN campaign. The difficulties in defining resistance and understanding its complexities don’t change the fact that it is a great concern and next generations nets should be considered in DRC to sustain effective malaria control.

## Supplementary information

**Additional file 1: Table S1.** Sites and periods where intensity assays were conducted and the history of LLIN distribution at provincial level.

## Data Availability

All data generated or analysed during this study are included in this article and are available from the corresponding author.

## Abbreviations

DRC: Democratic Republic of Congo
LLIN: Long lasting insecticidal net
PCR: Polymerase chain reaction
WHO: World Health Organization
CDC: Center for Disease Control
Kdr: Knock down resistance
NMCP: National Malaria Control Program
ANC: Ante-natal clinic
EPI: Expanded program of immunization
VGSC: Voltage-gated sodium channel
INRB: Institut National de Recherche Biomédicale
MFO: Mixed function oxidases
PBO: Piperonyl butoxide
