# Supplementary material for: Intensity of pyrethroid resistance in Anopheles gambiae before and after a mass distribution of insecticide-treated nets in Kinshasa and in 11 provinces of the Democratic Republic of Congo
Source: Malar J. 2020 Apr 30;19:169. doi: 10.1186/s12936-020-03240-6 (PMC7193383; doi:10.1186/s12936-020-03240-6)
Supplement: Supplementary file 1 — Additional file 1: Table S1. Sites and periods where intensity assays were conducted and the history of LLIN distribution at provincial level. [file 12936_2020_3240_MOESM1_ESM.docx]

Additional file 1

**Table S1.** Sites and periods where intensity assays were conducted and the history of LLIN distribution at provincial level**.**

| **Province** | **Site for larval collection** | **Years of data collection** | **GPS co-ordinates** | **LLIN mass campaign history by province** |
| --- | --- | --- | --- | --- |
| Kinshasa | Kimpoko | 2016-17 | -4.221112, 15.558783 | 2008, PermaNet 2.0 (deltamethrin)  2013, PermaNet 2.0 (deltamethrin)  2016, DawaPlus 2.0 (deltamethrin) |
|  | Bu | 2016-17 | -4.295485, 15.928586 |  |
|  | Kinkole | 2016-17 | -4.326610, 15.509998 |  |
|  | Kingasani | 2016-18 | -4.410647, 15.406118 |  |
| Kongo Central | Kasungulu | 2016-17 | -4.574124, 15.166611 | 2012, PermaNet 2.0 (deltamethrin)  2014, PermaNet 2.0 (deltamethrin)  2017, DawaPlus 2.0 &Yorkool (both deltamethrin) |
|  | Kimpese | 2018 | -5.574852, 14.428489 |  |
| Mai Ndombe | Inongo | 2017-18 | -1.919046, 18.934273 | 2010, Olyset (permethrin)  2015, Dawaplus 2.0 (deltamethrin), Olyset (permethrin), Magnet (alpha-cypermethrin), Yorkool (deltamethrin) |
| Kasai Central | Mikalayi | 2017-18 | -6.013150, 22.323417 | 2011, PermaNet 2.0 (deltamethrin)  2014, PermaNet 2.0 (deltamethrin) &Olyset (permethrin)  2017, Duranet (alpha-cypermethrin) |
| Haut Katanga | Kapolowe | 2017-18 | -10.906717, 26.946333 | 2012, PermaNet 2.0 (deltamethrin)  2012, Olyset (permethrin)  2016, Dawaplus 2.0 (deltamethrin) |
| Tanganyika | Kalemie | 2017-18 | -5.919200, 29.187250 |  |
| Sud Kivu | Katana | 2017-18 | -2.221363, 28.833621 | 2012, PermaNet 2.0 (deltamethrin)  2012, Olyset (permethrin)  2012, Duranet (alpha-cypermethrin) |
| Tshopo | Kabondo (Kisangani) | 2017-18 | 0.504117, 25.221883 | 2009, PermaNet 2.0 (deltamethrin)  2014, PermaNet 2.0 (deltamethrin)  2018, DawaPlus 2.0 (deltamethrin) |
| Sankuru | Lodja | 2018 | -3.515674, 23.595250 | 2011, PermaNet 2.0 (deltamethrin)  2015, PermaNet 2.0 (deltamethrin)  2015, Duranet (alpha-cypermethrin)  2018, Yorkool&PermaNet 2.0 (bothdeltamethrin) |
| Haut Uele | Pawa | 2018 | 2.514800, 27.696775 | 2009, PermaNet 2.0 (deltamethrin)  2014, PermaNet 2.0 (deltamethrin)  2018, Dawaplus 2.0 (deltamethrin) |
| Nord Ubangi | Karawa | 2018 | 3.333992, 20.314312 | 2011, PermaNet 2.0 (deltamethrin)  2015/2016, Olyset (permethrin), Olyset Plus (permethrin+PBO), PermaNet 2.0 (deltamethrin), PermaNet 3.0 (deltamethrin+PBO) |
